# Editorial: Negative valence systems

**DOI:** 10.3389/fnsys.2022.1014745

**Published:** 2022-09-21

**Authors:** Jennifer A. Honeycutt, Jared W. Young, Alessandra Porcu, Marta Sabariego

**Affiliations:** ^1^Department of Psychology and Program in Neuroscience, Bowdoin College, Brunswick, ME, United States; ^2^Department of Psychiatry, University of California, San Diego, San Diego, CA, United States; ^3^Drug Discovery & Biomedical Sciences, College of Pharmacy, University of South Carolina, Columbia, SC, United States; ^4^Program in Neuroscience and Behavior, Mount Holyoke College, South Hadley, MA, United States

**Keywords:** threat, loss, frustrative non-reward, RDoC = research domain criteria, negative valence systems

Our understanding of mental health is constantly evolving. More important than ever is the need for interdisciplinary and translational tools to better understand underlying mechanisms of mental illness to develop targeted therapeutics. Translational research relies on model systems to bridge the gap between human experiences and investigation of putative mechanisms. The study of emotionality—including negative affective states—has been particularly challenging (Barroca et al., 2022). The complexity of classification systems for psychiatric diagnoses has led to disciplinary fragmentation, making it difficult to comprehensively and translationally investigate affective dysfunction (Nestler and Hyman, 2010; Young et al., 2017). Furthermore, there is a disparity in our knowledge regarding sex differences in disorders of negative affect (e.g., anxiety, depression) despite increased prevalence in women (Kessler et al., 1993; Weissman et al., 1993; Pigott, 2003). Both clinical (human) and preclinical (model systems) research has historically focused on males, to the detriment of patients and our understanding of how sex contributes to outcomes (Beery and Zucker, 2011; Shansky, 2019; Sugimoto et al., 2019). Therefore, it is imperative that mental health research—particularly negative valence systems—include sex as a biological variable.

To advance translational affective research, we must move past symptom-based diagnoses to those that better delineate affective frameworks across species. The Research Domain Criteria (RDoC) project constitutes a translational framework for psychopathology research initiated by the National Institute of Mental Health (NIMH) to overcome limitations emerging from using symptom-based diagnostics (Insel et al., 2010). Instead, RDoC is informed by genetics, neurobiology, and behavioral observation vs. clinical diagnoses, enabling the study of discrete and/or overlapping endophenotypes to increase translational validity (Anderzhanova et al., 2017). NIMH's RDoC domain of negative valence systems includes five constructs of negative affect including: acute threat (fear), potential threat (anxiety), sustained threat, loss, and frustrative non-reward (Cuthbert and Insel, 2013). Although classified separately, these constructs are largely interrelated. Within the realm of threat (acute, possible, sustained), organisms must evaluate spatial and temporal proximity of a threatening stimulus. Loss and frustrative non-reward can be characterized by reactions to situations involving deprivation, withdrawal, devaluation or inability to obtain motivationally significant rewards (Figure 1). Generally, all constructs are disrupted in individuals with affective dysfunction, although not necessarily at the same levels.

**Figure 1:**
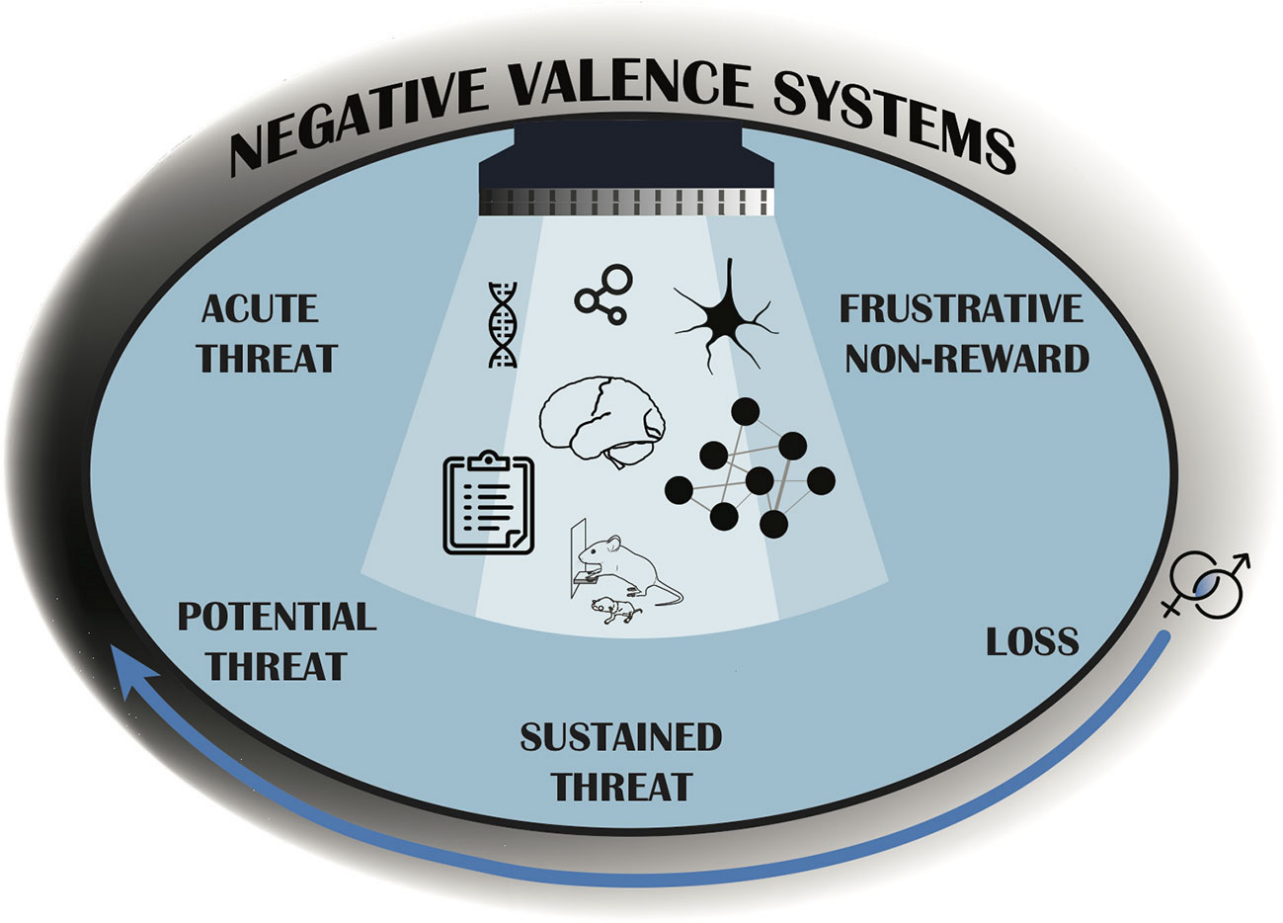
The Negative Valence Systems RDoC domain provides a structure for research that considers mental health and psychopathology in the context of neurobehavioral functioning, rather than diagnostic categories. Different aspects of the Negative Valence Systems domain are represented by five psychobiological dimensions, or constructs, which are studied along the full range of functioning from normal to abnormal (gray shading). The curved arrow represents that functioning changes across the life span, and so research on development that considers sex as a biological variable is essential. The seven images under the light represent examples of “Units of Analysis” which can be leveraged to investigate pathology and include: genetic, molecular, cellular, synaptic, physiological, behavioral, and self-report data sources. The RDoC framework encourages researchers to measure several units of analysis in order to more translationally understand the construct(s) under study (Antonis, 2020; Scidraw, 2020a,b; Tyler and Kravitz, 2020). These constructs should be considered within the context of the organisms environmental and social factors (denoted by blue background).

This timely Research Topic provides an opportunity for clinical and preclinical researchers to discuss the importance of investigating negative valence systems at multiple levels of analysis in humans and non-human models in a series of reviews and experimental reports.

Herein, Hu et al. highlight the importance of early life adversity (ELA) to pathology, discussing the development of negative valence circuits and caregiver psychosocial regulation in human infants and rodent models following typical rearing vs. ELA. The authors focus on the interface between the amygdala and mesolimbic dopamine system as a locus of dysfunction after ELA. Considering the importance of these circuits in social behavior and recruitment following ELA, the authors propose targeting these neural systems and upstream regions (i.e., habenula) to develop age-appropriate interventions following adversity.

Expanding on putative mechanisms underlying connectivity between negative experience and disease (Waters and Gould) review various ELA paradigms and describe different, yet interrelated, behavioral endophenotypes. The authors discuss the growing literature supporting adversity type, timing, duration, and intensity, likely contributing to mental health outcomes. They conclude that comparing brain changes resulting from different rodent ELA models will help clarify how specific subtypes of ELA influence discrete neural circuitry. While further studies are necessary, this knowledge could reveal specific targets for prevention/treatment of ELA-related neuropsychiatric disorders.

Specific to the construct of acute threat, du Plessis et al. investigated sex-differences in the neural networks that underlie threat conditioning in adult mice. Their work quantifies neural activity (via c-fos) across multiple brain regions after cued threat conditioning and found that trained females engaged prelimbic cortex, lateral amygdala, cortical amygdala, dorsal peduncular cortex, and subparafasicular nucleus more than—and subparaventricular zone less than—trained males. Using graph theory-derived analyses, they found sex differences in functional coordination of the threat conditioning network. Specifically, the globus pallidus and ventral lateral septum were the most robust hubs for trained males and females, respectively. These findings suggest the existence of sex differences in threat conditioning neural circuitry, which may at least partially explain sex differences in vulnerability to threat-related psychiatric disease.

Smith et al. propose that negatively valenced emotions, particularly anxiety, fear, and panic, are rooted in systems that evolved to deal with predation threat. Using the COVID-19 pandemic, the authors suggest a relatable story of the Predatory (or Threat) Imminence Continuum theory (PIC) (Fanselow and Lester, 1988; Bouton et al., 2001; Mobbs et al., 2007, 2015). The PIC proposes three defense modes where qualitatively distinct defensive behaviors change upon the perceived spatial, temporal, and psychological proximity to a life-threatening situation: pre-encounter, post-encounter, and circa-strike. Pre-encounter defense behaviors include increased vigilance and risk assessment when no specific threat is close. Early experiments simulating a naturalistic environment, meal pattern reorganization and cautiously leaving the nest area were observed in rodents (Fanselow et al., 1988), translated to people showing panic shopping and hoarding at the beginning of the pandemic. Once a threat is detected in close proximity, the dominant response is freezing, interpreted as akin to people avoiding leaving their houses during lockdown. Circa-strike responses (panic-like reactions observed in humans and rodents), take place when there is physical contact with a threat, not present in the RDoC Negative Valence System, and the authors suggest its addition be considered. It is worth noting that the COVID-19 pandemic could also be related to the constructs of loss and frustrative non reward since it led to sudden loss of significant sources of reinforcement via confinement, quarantine, social distance, economic loss, etc. (Vera-Villarroel, 2020), each having profound impacts on mental health (Serafini et al., 2020).

Another important mental health issue is the persistence of pathological fear memories. Thus, Maren more explicitly reflects on their durability and how their resistance precludes success of therapeutic interventions for disorders of fear and anxiety relying on extinction of conditioned responses. Maren reviews the literature regarding neural mechanisms underlying resistance to fear extinction, particularly when extinction procedures are administered soon after fear conditioning (Immediate Extinction Deficit, IED). Several studies reveal that IED is mediated by recruitment of a stress-related neural network that facilitates encoding and consolidation of fear memory even when the threat has passed. He further emphasizes the modulatory role that locus coeruleus norepinephrine exerts in amygdala-prefrontal cortical circuits and proposes specific neural mechanisms that balance excitation and inhibition in brain areas critical for extinction.

Overall, the reviews and studies presented here have important implications for understanding disorders of fear and anxiety in humans and using systems models. Deciphering the genetic, cellular, synaptic and behavioral mechanisms underlying negative valence systems remains critical for developing treatments to prevent/treat emotional problems in anxiety and stress-related disorders. However, in order to achieve this goal, researchers must harness the increased research validity that the RDoC Negative Valence Systems constructs afford to translational research models to better characterize and predict underlying neurobiological drivers of affective disorders.

## Author contributions

All authors listed have made a substantial, direct, and intellectual contribution to the work and approved it for publication.

## Funding

JH was supported by the Maine IDeA Network for Biomedical Excellence (INBRE) subaward, which is supported by the National Institute of General Medical Sciences of the National Institute of Health (Award P20GM103423). AP was supported by the National Center for Complementary and Integrative Health (Award K99AT010903). MS was supported by Mount Holyoke College, Program in Neuroscience and Behavior.

## Conflict of interest

The authors declare that the research was conducted in the absence of any commercial or financial relationships that could be construed as a potential conflict of interest.

## Publisher's note

All claims expressed in this article are solely those of the authors and do not necessarily represent those of their affiliated organizations, or those of the publisher, the editors and the reviewers. Any product that may be evaluated in this article, or claim that may be made by its manufacturer, is not guaranteed or endorsed by the publisher.
